# Lining the nest with more feathers increases offspring recruitment probability: Selection on an extended phenotype in the blue tit

**DOI:** 10.1002/ece3.6931

**Published:** 2020-10-22

**Authors:** Pauliina Järvinen, Jon E. Brommer

**Affiliations:** ^1^ Department of Biology University of Turku Turku Finland; ^2^ NOVIA University of Applied Sciences Ekenäs Finland

**Keywords:** extended phenotype, nest composition, nest construction, recruitment, selection, within‐species variation

## Abstract

Birds, among various other taxa, construct nests. Nests form an extended phenotype of the individual building it. Nests are used to extend control over the conditions in which offspring develop, and are therefore commonly considered to be shaped by selection. Nevertheless, scarcely any scientific evidence exist that nest composition is under selection. Here, we demonstrate with data from over 400 blue tit (*Cyanistes caeruleus*) nests collected over 8 years that a higher proportion of feathers in the nest lining is positively associated with the probability of offspring to recruit as a breeding adult later in life. Strikingly, the extended phenotype (nest) was associated stronger with recruitment probability than phenotypic traits that have typically been considered important in selection (laying date, and female size and condition). Our findings suggest that the choice of nest material could be a maternal behavior with potential lifelong effects on her offspring.

## Graphical abstract

Blue tit nests show an amazing variation. Many nests consist mainly of hair (a,b,c); others integrate feathers in the nest lining (d,e). Some nests also contain ornamental feathers (circled in yellow). Our study shows that offspring raised in nests where the cup is lined with more feathers have a higher probability to breed as an adult later in life.

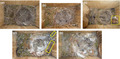

## 1 INTRODUCTION

Birds, among various other taxa, build nests to extend control over the conditions in which their offspring develop. The avian nest's main function is to allow efficient thermoregulation during incubation of the eggs and while the offspring are small (Deeming & Reynolds, 2015). Bird nests have a long evolutionary history and their thermoregulatory advantages may have been paramount to avian survival at the time when non‐avian dinosaurs went extinct (Hansell & Overhill, 2000). As vessels of reproduction, nests determine reproductive success. It therefore seems apparent that the composition of nests and their size is continuously being shaped by selection to provide optimal conditions for offspring survival.

Nest size has been positively related to reproductive parameters, such as clutch size (Álvarez & Barba, 2008; Soler, 2001), fledging (Alabrudzińska et al., 2003), and recruitment success (Álvarez & Barba, 2008, 2011), as well as to morphological traits (Mainwaring & Hartley, 2008), experience (Polo & Veiga, 2006), quality and condition of the builder (Mainwaring & Hartley, 2009; Tomás et al., 2006). Nest size also appears to be an individual‐specific trait; it shows significant between‐individual variation in female blue tits (*Cyanistes caeruleus*; Tomás et al., 2006; Järvinen, Kluen, Brommer, 2017), female pied flycatchers (*Ficedula hypoleuca*; Moreno et al., 2008), male southern masked weaverbirds (*Ploceus velatus*; Walsh et al., 2009), and male barn swallows (*Hirundo rustica*; Møller, 2005). Apart from its size, the components used to construct a nest have been shown to be related to the condition of the builder (Sergio et al., 2011), as well as the abundance of ectoparasites in the nest (Suarez‐Rodriguez et al., 2012; Tomás et al., 2012), recruitment success (Polo et al., 2015), nestling survival (Veiga & Polo, 2011), and physiology (Soler et al., 2017). To conclude, an ample amount of evidence exists that hints at the adaptive potential of nest construction, but few studies have demonstrated a long‐term selective benefit of certain nest types over others.

**FIGURE 1:**
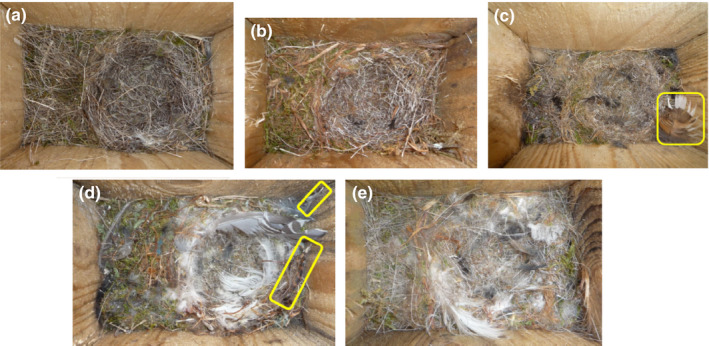
Pictures of blue tit nests taken from directly above when the nestlings are 2 days old (nestlings were temporarily removed). A blue tit nest has a base layer mainly consisting of moss into which a cup is formed on the side opposite of the nest opening (this opening is situated on the left‐hand side in each picture). The nest cup is lined with various materials and eggs are laid and incubated in this nest cup. Blue tit nests can have so‐called ornamental feathers, which are feathers that are on top of the nest, not integrated into the other material of the nest lining, and are outside the nest cup and therefore not in contact with nestlings. Ornamental feathers are indicated with yellow frames in the pictures. Blue tit nests show variability in their composition, which can be characterized along a gradient of increasing proportion of feathers in the nest lining. Pictures show: (a) A common nest type which consists mainly of ungulate hairs without feathers in the nest lining. (b) A nest with the lining composed of ungulate hair as well as strips of bark. (c) A nest with a low proportion of feathers in nest lining, as well as an ornamental feather. (d) Nest with a high proportion of feathers in the nest lining and ornamental feathers. (e) Nest with a high proportion of feathers

In this paper, we study a wild population of blue tits. The blue tit is a widely distributed and socially monogamous hole‐nesting passerine. It readily builds a nest in a nest box if available. Female blue tits choose the nesting site and build the nest that typically consists of moss, grass and other plant material, as well as hair, fur, wool and feathers (Figure 1; Britt & Deeming, 2011). The propensity to use feathers in the nest lining as well as nest height are traits of the female blue tit: these nest characteristics are repeatable and modestly heritable in female blue tits, but not in male blue tits (Järvinen, Kluen, Brommer, 2017; Järvinen, Kluen, Tiiri, et al., 2017). In our study population, about a quarter of nests have one or more ornamental feathers, which are often bright colored feathers that are placed on top of the nest outside the rim of the nest cup and therefore presumably have no role in terms of thermoregulation (Järvinen & Brommer, 2020; Figure 1). Work in a Spanish population of blue tits found that males are involved in ornamental feathering of nests and, if ornamental feathers are experimentally increased, males remove these (Sanz & García‐Navas, 2010; García‐Navas et al., 2013, see also Mainwaring et al., 2016). However, the number of ornamental feathers in a nest is repeatable in our study population only in females, but not in males (Järvinen & Brommer, 2020). Because repeatability is the upper limit of heritability, non‐repeatable traits are not inherited by descendants and are therefore unable to respond to selection (Falconer & MacKay, 1996). Thus, we here quantify selection on blue tit nest characteristics from the perspective that the nest is an extended phenotype of the female that has constructed it. In particular, we study whether the height of her nest, proportion of feathers she used in the nest lining and whether or not there are ornamental feathers on the nest are associated with reproductive success in terms of the probability of an offspring to survive to fledging and the probability of a fledgling to recruit back into the local breeding population. In doing so, we recognize that other female traits such as when she has produced her first egg in the season (laying date) and her size and mass may be confounded with both her extended phenotype and her reproductive success. To this end, we perform multiple regression analyses where we also include these covariates to statistically correct for their potentially confounding effect.

## 2 MATERIAL AND METHODS

### 2.1 Study site

The study site was located in Tammisaari, southwest Finland. The site has been the location of a long‐term study of blue tits since 2005 and consisted of 319–363 nest boxes during the study period of 2012–2019. The site spans over 10 km^2^ and consists of mixed boreal forest that is interspersed with arable land.

### 2.2 Data collection

We used data on first broods collected in each breeding season between 2012 and 2019. During these 8 years, no experimental manipulations were performed. We determined laying dates by weekly nest box checks that we ran from late April until the first eggs hatched. We calculated the laying date of an incomplete clutch with the assumption that one egg was laid per day. We determined the hatching date (day 0) by daily nest checks starting on the expected date of hatching as described by Kluen et al. (2011).

### 2.3 Nest characteristics

On day 2, we measured the nests for height (see Järvinen, Kluen, Brommer, 2017; Järvinen, Kluen, Tiiri, et al., 2017), temporarily removed the nestlings and photographed the nest (as shown in Figure 1). We then temporally removed the nest from the nest box. By careful partial de‐construction of the nest, the proportion of the different materials used to construct the nest was quantified by eye. A blue tit nest has a thick layer typically consisting mainly of moss as its base. On top of this base layer, a nest cup is constructed from different materials (mainly using hair of ungulates, mammalian fur, feathers, grass, and strips of bark; Figure 1). The proportion of feathers in the nest lining was computed as the proportion of feathers in the nest after excluding moss because moss is practically only used in the base layer whose thickness may vary substantially (see Järvinen, Kluen, Brommer, 2017; Järvinen, Kluen, Tiiri, et al., 2017). Using the photographs, a single observer (author PJ) identified nest feather ornaments. Feathers were considered ornamental when they were placed on top of the nest, not in contact with nestlings, and stood out from the bulk of the material based on a large size or contrasting color (Järvinen & Brommer, 2020.; illustrated in Figure 1). As most (76%) of the nests in our population do not contain nest feather ornaments (Järvinen & Brommer, 2020), ornamentation of the nest was included as a binary variable denoting whether there were feather ornaments (yes), or not (no).

### 2.4 Parental identity and traits

When the nestlings were around 5–9 days old, we trapped parents in the nest box when they came in to feed their offspring. We ringed each parent with a metal ring to allow individual identification. We measured the body mass with a spring balance to the nearest 0.1 g and the tarsus with a sliding calliper to the nearest 0.1 mm.

### 2.5 Fledging status

We included all nests with nestlings alive on day 2 (when the nest construction was scored) in the data. Nestlings were ringed when they were 9 days old with a metal ring to allow individual identification. The presence or absence of nestlings in each nest was established when they were 16 days old at which age they have attained the skeletal size of adults. Blue tits fledge when 18 days old or older. We visited all nests again after the nestlings had fledged, and any nestlings that were not found dead in the nest box at that time were assumed to have fledged successfully because parents do not remove fully grown nestlings (i.e., nestling older than 16 days).

### 2.6 Statistical analysis

All explanatory variables were standardized to zero mean and unit standard deviation to facilitate comparison of their effect sizes. All analyses were conducted in R (R Core Team, 2019). We summarize the statistical analyses and sample sizes in Table 1.

**TABLE 1:** A list and description as well as sample sizes of the data for each GLMM

	Variable	Type/transformation	Description/scale	*n*	Model
Response	Fledging probability	Binomial (success/failure)	Nr of success/failure out of hatchlings per nest	656	F
	Recruitment probability	Binomial (success/failure)	Nr of success/failure out of fledglings per nest	403	R
Fixed effect	Nest height	Standardized	Height (36.3–155 mm)		F,R
	Proportion of feathers	Standardized arcsine‐square‐root‐transformed	Proportion of nest lining that are feathers (0–1)		F,R
	Nest ornamentation	Factorial	No, yes		F,R
	Lay date	Standardized	April days (20–57)		F,R
	Female body mass	Standardized	Mean mass per female/season (9.7–13.8 g)		F,R
	Female tarsus length	Standardized	Mean length per female/season (15.0–18.3 mm)		F, R
Random effect	Year	Factorial effect	2012–2019	8	F
			2012–2017	6	R
	Individual	Factorial effect	Female ID	431	F
			Female ID	285	R

Note

The column “Variable” denotes the response, fixed and random effect variables. Under “Type/transformation,” we specify for the response variables which type of errors the GLMM used and for the fixed and random effects whether the variable was factorial or continuous, where “standardized” refers to when the *Z*‐score of a continuous variable was used in the analysis. Under “Description/scale,” a verbal explanation is provided as well as the range of values in the data for fixed effects. The “*n*” provided for the response and random effects refers to the number of nests and the number of levels, respectively. The “Model” referred to as “F” and “R” are the analysis of the probability of a hatchling to fledge and of a fledgling to recruit, respectively.

We constructed Generalized Linear Mixed Models (GLMM) with binomial response variables and R package “lme4” (Bates et al., 2015) to measure the effect of nest characteristics on blue tit reproductive success. Our data were non‐independent with repeated measures of females and years and we included these as random effects. We ran separate models with two different measures of reproductive success as response variables: fledging and local recruitment probability. Fledging probability describes the proportion of offspring that successfully fledged out of a completed clutch between 2012 and 2019. Local recruitment success describes the proportion of fledglings that returned to one of our nest boxes to breed later in life. We measured local recruitment of individuals that hatched between 2012 and 2017 to allow a minimum of 2 years for recruitment. Blue tits are short‐lived birds and most individuals have recruited before their second year of life (Stenning, 2018). Local recruitment probability represents a minimum probability to produce a reproducing offspring, because fledglings may also recruit to breed outside our box network.

The focus of our analysis is on inferring the association of nest characteristics to reproductive success. Because these nest characteristics may covary with other aspects that affect reproductive success, we statistically controlled for a number of potential other aspects by including these as fixed effects in the models. We included laying date given its association with avian reproductive success (Nilsson, 1994). To control for a potential effect of female condition on reproductive success, we included female tarsus length and body mass as explanatory variables in both models, which is statistically equivalent to using tarsus‐corrected (or residual) body mass. Lastly, we included female age (1 vs. ≥2 years old) as a fixed effect because experience has been shown to affect reproductive performance and nest composition (Muth & Healy, 2011, 2014). Because earlier analyses showed that nest height, the proportion of feathers and feather nest ornaments were not repeatable in males (Järvinen, Kluen, Brommer, 2017; Järvinen & Brommer, 2020), we did not consider male traits in these analyses.

## 3 RESULTS

The overall probability of a hatchling surviving to fledge was 68.5% (4,748/6,930). Local recruitment probability into the breeding population of a fledgling was on average 5.7% (178/3,096). Of the 656 nests, 26% (173/656) contained one or more ornamental feathers.

**FIGURE 2:**
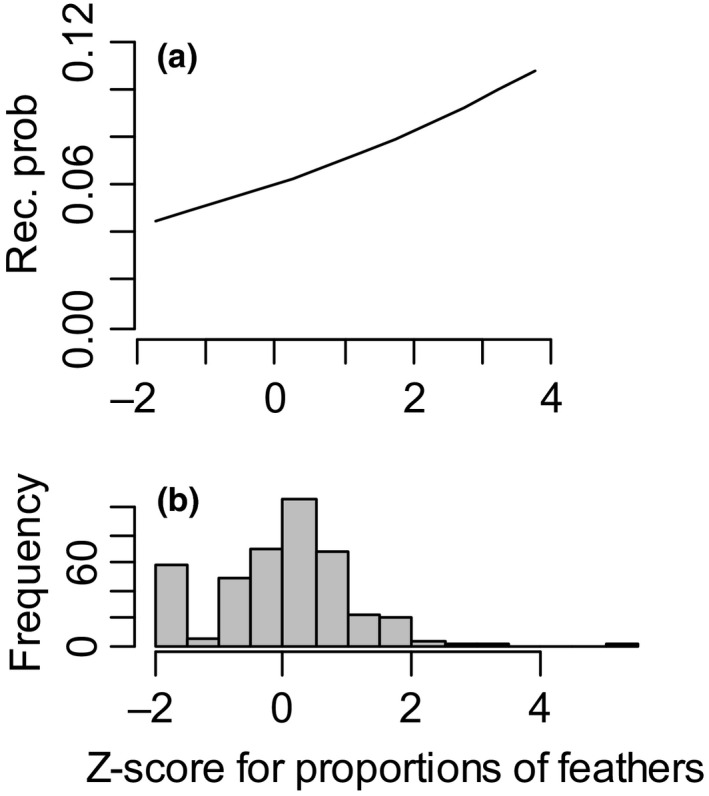
(a) Probability of a fledgling to recruit locally into the breeding population (Rec. prob.) as a function of the proportion of feathers in the nest lining (standardized *Z*‐scores). (b) Distribution of the standardized proportion of feathers in the nest lining for the data used to analyze the recruitment probability (Table 1)

Nest characteristics (the nest height, proportion of feathers in the nest lining, and presence/absence of feather nest ornaments) had no effect on fledging probability (Table 2). The proportion of feathers in the nest lining had a significant positive effect on recruitment probability (Table 2; Figure 2). The female morphological measures (body mass and tarsus length) did not affect recruitment probability of fledglings but female tarsus length had a significant positive and female body mass a significant negative effect on survival probability from hatching to fledging (Table 2).

**TABLE 2:** Results of a binomial GLMM of nest and female characteristics on the proportion of young fledged (fledging probability) and the proportion of fledglings that recruited into the breeding population later in life (recruitment probability)

	Fledging probability				Recruitment probability			
	Variance				Variance			
Random effects								
Female ID	3.02				0.25			
Year	1.24				0.18			
	**Est.**	***SE***	***Z***	***p* > *z***	**Est.**	***SE***	***Z***	***p* > *z***
Fixed effects								
(Intercept)	**1.29**	**0.41**	**3.15**	**0.002**	**−2.94**	**0.23**	**−12.8**	**<0.001**
Nest height	−0.018	0.069	0.26	0.79	0.067	0.090	0.74	0.46
Feather %	0.059	0.060	0.98	0.33	**0.18**	**0.081**	**2.16**	**0.031**
Ornament	0.18	0.12	1.46	0.14	−0.12	0.19	0.61	0.54
Lay date	−0.025	0.077	0.33	0.74	−0.080	0.097	0.83	0.41
Mass ♀	**−0.31**	**0.071**	**−4.33**	**<0.001**	0.11	0.10	1.09	0.28
Tarsus ♀	**0.30**	**0.088**	**3.48**	**<0.001**	−0.10	0.093	1.09	0.28
Age♀ (year)	−0.17	0.11	1.44	0.15	−0.23	0.17	1.35	0.18

Note

The statistically significant (*p* < 0.05) variables are shown in bold. Sample sizes presented in Table 1.

## 4 DISCUSSION

A higher proportion of feathers used in the lining of blue tit nests is associated with an increase in the probability that a fledgling recruits back into the local breeding population. Strikingly, only the proportion of feathers in the nest lining is associated with offspring local recruitment probability, whereas laying date and variables capturing the female's somatic condition (tarsus and mass) and her age were not. In contrast, we find no association between nest characteristics and survival during the nestling period (hatchling to fledgling). Taken together, these findings imply that selection on nest composition primarily acts via long‐term (i.e., post‐fledging) fitness benefits to offspring.

There are a number of possible non‐mutually exclusive pathways by which feathers in the nest lining can provide direct long‐term benefits for offspring performance. Because the main benefit of feathers is their improved thermal insulation of the nest (Dawson et al., 2011; Hilton et al., 2004; Lombardo, 1995; Møller, 1984; Pinowski et al., 2006; Windsor et al., 2013), one possibility is that this thermoregulatory advantage has long‐term fitness consequences for offspring. Feathers in the nest can also provide protection against microbial infections (Peralta‐Sanchez et al., 2010; Peralta‐Sánchez et al., 2013; Ruiz‐Castellano et al., 2016), and thereby provide long‐term fitness consequences. Indeed, Soler et al. (2017) showed by experimental manipulation that the amount of feathers in nests affected the probability of nestling survival by affecting their telomere attrition. One further possibility is that feathers in the nest protect nestlings against ectoparasites (López‐Rull & Macías, 2015). For example, Winkler (1993) showed that experimental removal of feathers from tree swallow (*Tachycineta bicolor*) nests caused offspring to be significantly higher infected with mites and lice and caused slower growth rate compared to the nestlings in control nests. Clearly, therefore, a number of potential pathways exist whereby a higher proportion of feathers in the nest lining could have a direct causal effect on offspring performance.

In addition to the direct benefits of having more feathers in the nest lining for offspring post‐fledging performance, as described above, a greater proportion of feathers in the nest lining may also benefit nestling fitness indirectly. That is, the proportion of feathers in the nest lining—as an extended phenotype of the female that built it—could be associated with the female's capacity to produce offspring with a high probability to recruit. For example, a nest with a high proportion of feathers may, because of its superior thermoregulatory capacity during incubation, reduce the energetic costs of incubation to the female and allow her to produce offspring with a higher recruitment probability. Another possibility is that only females of superior quality build nests with more feathers in the nest lining and produce offspring with a higher recruitment probability. One example of an association between a nest characteristic and female blue tits’ individual quality is that females infected with a higher amount of blood parasites built lighter nests (Tomás et al., 2006). To statistically control for such confounding, we included in our analyses the female's age, and two somatic aspects (tarsus length and body mass), but clearly other unmeasured aspects of female health could covary with the proportion of feathers in the nest and affect offspring recruitment probability. Apart from nest characteristics potentially being associated with fitness aspects of the female that built it, the nest itself may signal its potential for direct benefits to the offspring's sire, assuming having more feathers in the nest lining indeed causally affects offspring performance. As a response to the potential direct benefits for his offspring provided by a nest with more feathers in the nest lining, a male may upregulate his paternal effort. Hence, the direct and indirect effects of having more feathers in the nest lining are not mutually exclusive, and both may act to improve offspring recruitment probability. Because our findings are based on association, careful experimental studies are needed to establish whether there are causal links between the proportion of feathers in the nest lining and offspring recruitment probability or not.

An intriguing aspect of our study is that any fitness advantages of nest composition are not apparent during the period offspring stay in the nest. We found that nestlings produced by females with a longer tarsus (measure of skeletal size) have a higher probability to fledge, indicating that larger females are more successful during the nestling period in this population. In general, directional selection for larger size is common (Kingsolver & Pfennig, 2004; Morrissey & Hadfield, 2011). More surprisingly, the offspring of females in better somatic condition (as measured by body mass) had a lowered probability to survive to fledging. This could be an example of differential investment, where a female balances investment in herself versus current progeny and greater investment in own somatic condition, at the expense of nestling survival, may benefit reproduction in the next breeding season (Karell et al., 2009).

As for any trait showing an association with fitness based on descriptive data, experimental manipulation (of, in our case, the proportion of feathers in the nest lining) is required to determine whether the proportion of feathers in the nest lining is itself indeed the target of selection or simply a correlate of another trait determining recruitment probability of offspring. Nevertheless, because a part of the variation in nest composition is inherited from mother to daughter (Järvinen, Kluen, Brommer, 2017; Järvinen, Kluen, Tiiri, et al., 2017), our finding that variation in nest composition is associated with reproductive fitness suggests that nest construction in blue tits has ecological relevance and is an adaptive behavior with potential to evolve in response to environmental change.

## CONFLICT OF INTEREST

The authors declare they have no conflict of interest.

## AUTHOR CONTRIBUTIONS

**Pauliina Järvinen:** Data curation (equal); formal analysis (equal). **Jon E. Brommer:** Conceptualization (equal); data curation (lead); formal analysis (equal); funding acquisition (lead); project administration (lead); supervision (lead).

## Data Availability

The datasets generated and analyzed during the current study are available in the Dryad repository under https://doi.org/10.5061/dryad.3ffbg79gj.

## References

[ece36931-bib-0001] Alabrudzińska, J. , Kaliński, A. , Słomczyński, R. , Wawrzyniak, J. , Zieliński, P. , & Bańbura, J. (2003). Effects of nest characteristics on breeding success of great tits *Parus major* . Acta Ornithologica, 38(2), 151–154. 10.3161/068.038.0202

[ece36931-bib-0002] Álvarez, E. , & Barba, E. (2008). Nest quality in relation to adult bird condition and its impact on reproduction in great tits *Parus major* . Acta Ornithologica, 43(1), 3–9. 10.3161/000164508X345275

[ece36931-bib-0003] Álvarez, E. , & Barba, E. (2011). Nest characteristics and reproductive performance in great tits *Parus major* . Ardeola, 58(1), 125–136. 10.13157/arla.58.1.2011.125

[ece36931-bib-0005] Bates, D. , Mächler, M. , Bolker, B. , & Walker, S. (2015). Fitting linear mixed‐effects models using lme4. Journal of Statistical Software, 67(1), 1–48. 10.18637/jss.v067.i01

[ece36931-bib-0006] Britt, J. , & Deeming, D. C. (2011). First‐egg date and air temperature affect nest construction in blue tits *Cyanistes caeruleus*, but not in great tits *Parus major* . Bird Study, 58(1), 78–89. 10.1080/00063657.2010.524916

[ece36931-bib-0008] Dawson, R. D. , O'Brien, E. L. , & Mlynowski, T. J. (2011). The price of insulation: Costs and benefits of feather delivery to nests for male tree swallows *Tachycineta bicolor* . Journal of Avian Biology, 42(2), 93–102. 10.1111/j.1600-048X.2010.05208.x

[ece36931-bib-0009] Deeming, D. C. , & Reynolds, S. J. (Eds.) (2015). Nest, eggs, and incubation: New ideas about avian reproduction. Oxford Univ. Press.

[ece36931-bib-0010] Falconer, D. S. , & Mackay, T. F. C. (1996). Introduction to quantitative genetics (4th ed.). Longman.

[ece36931-bib-0012] García‐Navas, V. , Ortego, J. , Ferrer, E. S. , & Sanz, J. J. (2013). Feathers, suspicions, and infidelities: An experimental study on parental care and certainty of paternity in the blue tit. Biological Journal of the Linnean Society, 109(3), 552–561. 10.1111/bij.12079

[ece36931-bib-0013] Hansell, M. , & Overhill, R. (2000). Bird nests and construction behaviour. Cambridge University Press 10.1017/CBO9781139106788

[ece36931-bib-0014] Hilton, G. M. , Hansell, M. H. , Ruxton, G. D. , Reid, J. M. , & Monaghan, P. (2004). Using artificial nests to test importance of nesting material and nest shelter for incubation energetics. The Auk, 121(3), 777 10.1642/0004-8038(2004)121[0777:UANTTI]2.0.CO;2

[ece36931-bib-0015] Järvinen, P. , & Brommer, J. E. (2020). Nest ornaments and feather composition form an extended phenotype syndrome in a wild bird. Behavioral Ecology and Sociobiology, 74, 134 10.1007/s00265-020-0291

[ece36931-bib-0016] Järvinen, P. , Kluen, E. , & Brommer, J. E. (2017). Low heritability of nest construction in a wild bird. Biology Letters, 13(10), 20170246 10.1098/rsbl.2017.0246 29046371 PMC5665766

[ece36931-bib-0017] Järvinen, P. H. , Kluen, E. , Tiiri, M. , & Brommer, J. E. (2017). Experimental manipulation of Blue Tit nest height does not support the thermoregulation hypothesis. Ornis Fennica, 94(2), 82–92.

[ece36931-bib-0018] Karell, P. , Pietiäinen, H. , Siitari, H. , Pihlaja, T. , Kontiainen, P. , & Brommer, J. E. (2009). Parental allocation of additional food to own health and offspring growth in a variable environment. Canadian Journal of Zoology, 87(1), 8–19. 10.1139/Z08-133

[ece36931-bib-0019] Kingsolver, J. G. , & Pfennig, D. W. (2004). Individual‐level selection as a cause of cope's rule of phyletic size increase. Evolution, 58, 1608–1612. 10.1111/j.0014-3820.2004.tb01740.x 15341162

[ece36931-bib-0020] Kluen, E. , de Heij, M. E. , & Brommer, J. E. (2011). Adjusting the timing of hatching to changing environmental conditions has fitness costs in blue tits. Behavioral Ecology and Sociobiology, 65(11), 2091–2103. 10.1007/s00265-011-1218-y

[ece36931-bib-0021] Lombardo, M. P. (1995). Effect of feathers as nest insulation on incubation behavior and reproductive performance of tree swallows (*Tachycineta bicolor*). The Auk, 112(4), 973–981. 10.2307/4089028

[ece36931-bib-0022] López‐Rull, I. , & Macías, G. C. (2015). Control of invertebrate occupants of nests In Deeming D. C. & Reynolds S. J. (Eds.), Nests, eggs, and incubation (pp. 82–96). Oxford: Oxford University Press 10.1093/acprof:oso/9780198718666.003.0008

[ece36931-bib-0023] Mainwaring, M. C. , & Hartley, I. R. (2008). Seasonal adjustments in nest cup lining in blue tits *Cyanistes caeruleus* . Ardea, 96(2), 278–282. 10.5253/078.096.0213

[ece36931-bib-0024] Mainwaring, M. C. , & Hartley, I. R. (2009). Experimental evidence for state‐dependent nest weight in the blue tit, *Cyanistes caeruleus* . Behavioural Processes, 81(1), 144–146. 10.1016/j.beproc.2009.02.001 19429209

[ece36931-bib-0025] Mainwaring, M. C. , Wolfenden, A. , Read, J. E. , Robson, J. M. A. , Tomlinson, C. J. , & Hartley, I. R. (2016). Feathering the nest: The effects of feather supplementation to Blue Tit nests. Avian Biology Research, 9(2), 89–95. 10.3184/175815516X14551240159329

[ece36931-bib-0026] Møller, A. P. (1984). On the use of feathers in birds' nests: Predictions and tests. Ornis Scandinavica, 15(1), 38–42. 10.2307/3676000

[ece36931-bib-0027] Møller, A. P. (2006). Rapid change in nest size of a bird related to change in a secondary sexual character. Behavioral Ecology, 17(1), 108–116. 10.1093/beheco/arj003

[ece36931-bib-0029] Moreno, J. , Martínez, J. , Corral, C. , Lobato, E. , Merino, S. , Morales, J. , Martínez‐De La Puente, J. , & Tomás, G. (2008). Nest construction rate and stress in female pied flycatchers *Ficedula hypoleuca* . Acta Ornithologica, 43(1), 57–64. 10.3161/000164508X345338

[ece36931-bib-0030] Morrissey, M. B. , & Hadfield, J. D. (2011). Directional selection in temporally replicated studies is remarkably consistent. Evolution, 66–2, 435–442. 10.1111/j.1558-5646.2011.01444.x 22276539

[ece36931-bib-0031] Muth, F. , & Healy, S. D. (2011). The role of adult experience in nest building in the zebra finch, *Taeniopygia guttata* . Animal Behaviour, 82(2), 185–189. 10.1016/j.anbehav.2011.04.021

[ece36931-bib-0032] Muth, F. , & Healy, S. D. (2014). Zebra finches select nest material appropriate for a building task. Animal Behaviour, 90, 237–244. 10.1016/j.anbehav.2014.02.008

[ece36931-bib-0033] Nilsson, J.‐Å. (1994). Energetic bottle‐necks during breeding and the reproductive cost of being too early. Journal of Animal Ecology, 63, 200–208. 10.2307/5595

[ece36931-bib-0034] Peralta‐Sanchez, J. M. , Møller, A. P. , Martin‐Platero, A. M. , & Soler, J. J. (2010). Number and colour composition of nest lining feathers predict eggshell bacterial community in barn swallow nests: An experimental study. Functional Ecology, 24(2), 426–433. 10.1111/j.1365-2435.2009.01669.x

[ece36931-bib-0035] Peralta‐Sánchez, J. M. , Soler, J. J. , Martín‐Platero, A. M. , Knight, R. , Martínez‐Bueno, M. , & Møller, A. P. (2014). Eggshell bacterial load is related to antimicrobial properties of feathers lining barn swallow nests. Microbial Ecology, 67(2), 480–487. 10.1007/s00248-013-0338-5 24317898

[ece36931-bib-0036] Pinowski, J. , Haman, A. , Jerzak, L. , Pinowska, B. , Barkowska, M. , Grodzki, A. , & Haman, K. (2006). The thermal properties of some nests of the Eurasian Tree Sparrow *Passer montanus* . Journal of Thermal Biology, 31(7), 573–581. 10.1016/j.jtherbio.2006.05.007

[ece36931-bib-0037] Polo, V. , Rubalcaba, J. G. , & Veiga, J. P. (2015). Green plants in nests reduce offspring recruitment rates in the spotless starling. Behavioral Ecology, 26(4), 1131–1137. 10.1093/beheco/arv056

[ece36931-bib-0038] Polo, V. , & Veiga, J. P. (2006). Nest ornamentation by female spotless starlings in response to a male display: An experimental study. Journal of Animal Ecology, 75(4), 942–947. 10.1111/j.1365-2656.2006.01103.x 17009757

[ece36931-bib-0039] R Core Team. R (2019). A language and environment for statistical computing. R Foundation for Statistical Computing Retrieved from https://www.R‐project.org/

[ece36931-bib-0040] Ruiz‐Castellano, C. , Tomás, G. , Ruiz‐Rodríguez, M. , Martín‐Gálvez, D. , & Soler, J. J. (2016). Nest material shapes eggs bacterial environment. PLoS One, 11(2), e0148894 10.1371/journal.pone.0148894 26871451 PMC4752222

[ece36931-bib-0041] Sanz, J. J. , & García‐Navas, V. (2011). Nest ornamentation in blue tits: Is feather carrying ability a male status signal? Behavioral Ecology, 22(2), 240–247. 10.1093/beheco/arq199

[ece36931-bib-0042] Sergio, F. , Blas, J. , Blanco, G. , Tanferna, A. , Lopez, L. , Lemus, J. A. , & Hiraldo, F. (2011). Raptor nest decorations are a reliable threat against conspecifics. Science, 331(6015), 327–330. 10.1126/science.1199422 21252345

[ece36931-bib-0043] Soler, J. J. (2001). Nest size affects clutch size and the start of incubation in magpies: An experimental study. Behavioral Ecology, 12(3), 301–307. 10.1093/beheco/12.3.301

[ece36931-bib-0044] Soler, J. J. , Ruiz‐Castellano, C. , Figuerola, J. , Martín‐Vivaldi, M. , Martínez‐de la Puente, J. , Ruiz‐Rodríguez, M. , & Tomás, G. (2017). Telomere length and dynamics of spotless starling nestlings depend on nest‐building materials used by parents. Animal Behaviour, 126, 89–100. 10.1016/j.anbehav.2017.01.018

[ece36931-bib-0045] Stenning, M. (2018). The blue tit. Bloomsbury Publishing.

[ece36931-bib-0046] Suarez‐Rodriguez, M. , Lopez‐Rull, I. , & Macias, G. C. (2013). Incorporation of cigarette butts into nests reduces nest ectoparasite load in urban birds: New ingredients for an old recipe? Biology Letters, 9(1), 20120931 10.1098/rsbl.2012.0931 23221874 PMC3565511

[ece36931-bib-0047] Tomás, G. , Merino, S. , Martínez‐de la Puente, J. , Moreno, J. , Morales, J. , Lobato, E. , Rivero‐de Aguilar, J. , & del Cerro, S. (2012). Interacting effects of aromatic plants and female age on nest‐dwelling ectoparasites and blood‐sucking flies in avian nests. Behavioural Processes, 90(2), 246–253. 10.1016/j.beproc.2012.02.003 22387676

[ece36931-bib-0049] Tomás, G. , Merino, S. , Moreno, J. , Sanz, J. J. , Morales, J. , & García‐Fraile, S. (2006). Nest weight and female health in the blue tit (*Cyanistes caeruleus*) (Peso del Nido y Estado de Salud de la Hembra en el *Cyanistes caeruleus*). The Auk, 123(4), 1013–1021. 10.2307/25150216

[ece36931-bib-0051] Veiga, J. P. , & Polo, V. (2011). Feathers in the spotless starling nests: A sexually selected trait? Behaviour, 148(11‐13), 1355–1371. 10.1163/000579511X608684

[ece36931-bib-0052] Walsh, P. T. , Hansell, M. , Borello, W. D. , & Healy, S. D. (2009). Repeatability of nest morphology in African weaver birds. Biology Letters, 6(2), 149–151. 10.1098/rsbl.2009.0664 19846449 PMC2865054

[ece36931-bib-0053] Windsor, R. L. , Fegely, J. L. , & Ardia, D. R. (2013). The effects of nest size and insulation on thermal properties of tree swallow nests. Journal of Avian Biology, 44(4), 305–310. 10.1111/j.1600-048X.2013.05768.x

[ece36931-bib-0054] Winkler, D. W. (1993). Use and importance of feathers as nest lining in Tree Swallows (*Tachycineta bicolor*). The Auk [internet]., 110(1), 29–36.

